# Supplementary material for: RIP1 autophosphorylation is promoted by mitochondrial ROS and is essential for RIP3 recruitment into necrosome
Source: Nat Commun. 2017 Feb 8;8:14329. doi: 10.1038/ncomms14329 (PMC5309790; doi:10.1038/ncomms14329)
Supplement: Supplementary Information — Supplementary Figures [file ncomms14329-s1.pdf]

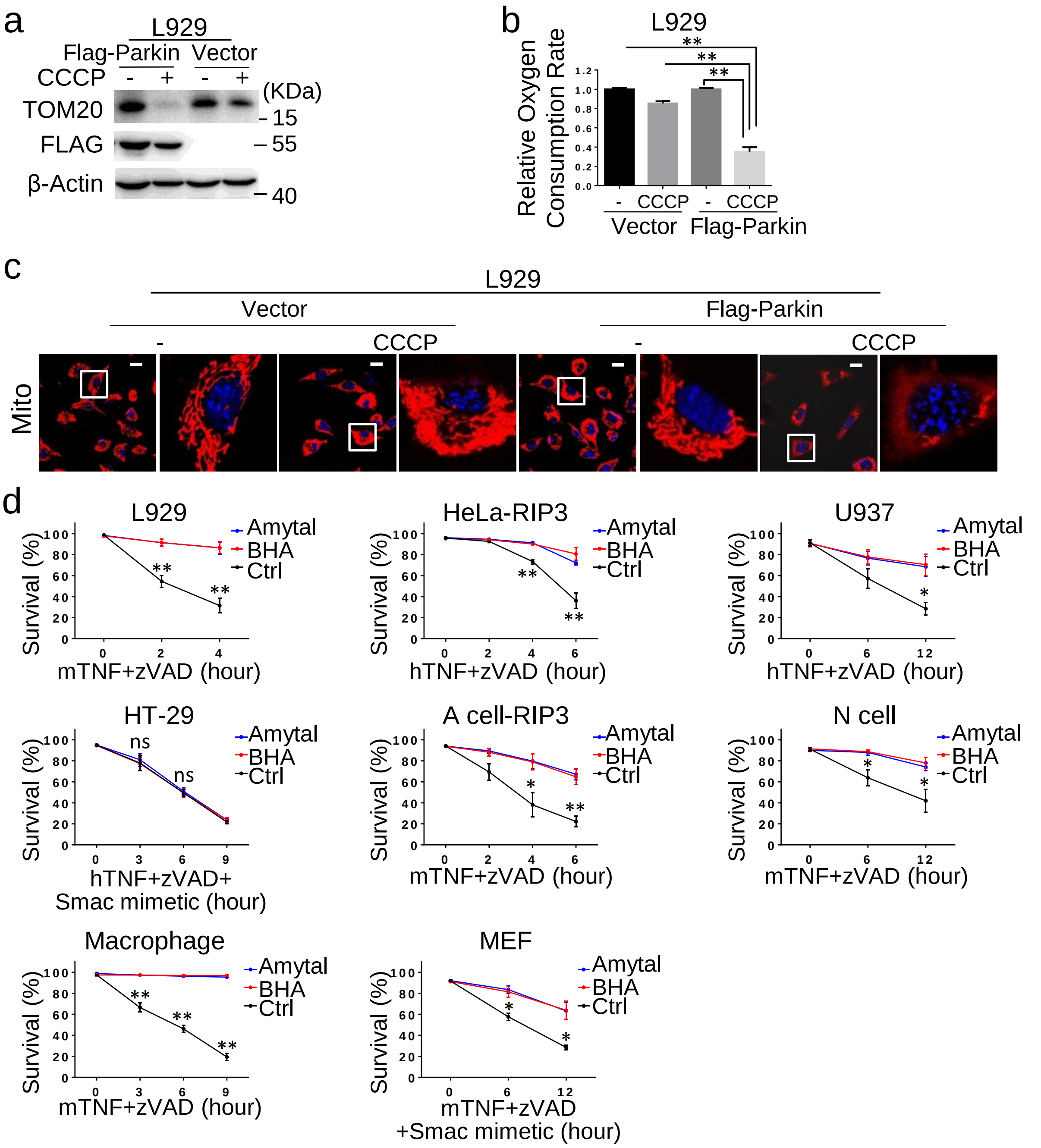
a
L929
Flag-Parkin Vector
CCCP - + - +
TOM20
FLAG
β-Actin
(KDa)
15
55
40
b
L929
Relative Oxygen Consumption Rate
- CCCP - CCCP
Vector Flag-Parkin
c
L929
Vector
Flag-Parkin
-
CCCP
Mito
d
L929
HeLa-RIP3
U937
HT-29
A cell-RIP3
N cell
Macrophage
MEF
Amytal
BHA
Ctrl
Survival (%)
mTNF+zVAD (hour)
hTNF+zVAD (hour)
hTNF+zVAD+ Smac mimetic (hour)
mTNF+zVAD +Smac mimetic (hour)
ns

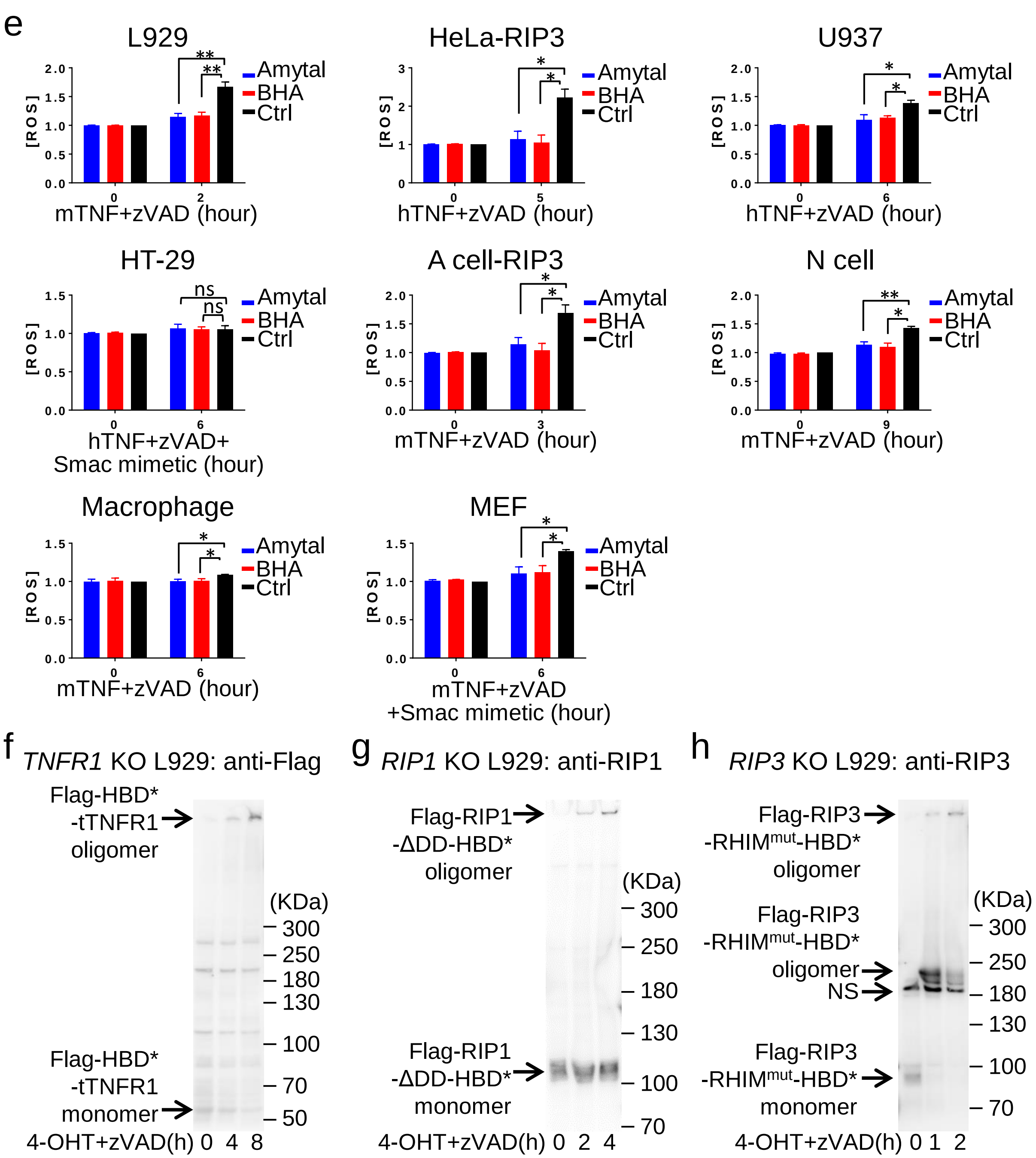
e
L929
HeLa-RIP3
U937
HT-29
A cell-RIP3
N cell
Macrophage
MEF
Amytal
BHA
Ctrl
[ROS]
mTNF+zVAD (hour)
hTNF+zVAD (hour)
hTNF+zVAD+
Smac mimetic (hour)
mTNF+zVAD
+Smac mimetic (hour)
ns
f
TNFR1 KO L929: anti-Flag
Flag-HBD*
-tTNFR1
oligomer
Flag-HBD*
-tTNFR1
monomer
(KDa)
4-OHT+zVAD(h) 0 4 8
g
RIP1 KO L929: anti-RIP1
Flag-RIP1
-ΔDD-HBD*
oligomer
Flag-RIP1
-ΔDD-HBD*
monomer
4-OHT+zVAD(h) 0 2 4
h
RIP3 KO L929: anti-RIP3
Flag-RIP3
-RHIMmut-HBD*
oligomer
Flag-RIP3
-RHIMmut-HBD*
monomer
NS
4-OHT+zVAD(h) 0 1 2

Supplementary Figure 1. ROS are involved in TNF-induced necroptosis of many but not all cells/cell lines, related to Figure 1.

a. Wildtype (WT) L929 cells transfected with Flag-Parkin expression vector or empty vector were treated with CCCP (10 μM) for 48 hours. These cells, together with corresponding CCCP non-treated control cells were subjected to Western blotting with anti-Tom20, anti-Flag, and anti-β-Actin antibodies. **b**. Relative oxygen consumption rates (OCR) of the cells described in (a) were measured by Seahorse XF96 analyzer (see Methods for details). Data are represented as means ± s.e.m of quadruplicate samples and are representative of two independent experiments. **c**. Cells described in (a) were stained with MitoTracker Red and then subjected to confocal microscopy. **d**. Viabilities of L929, HeLa-RIP3, U937, HT-29, A cell-RIP3, N cell, primary peritoneal macrophage and MEF were determined after mTNF+zVAD or human TNF (hTNF)+zVAD+/-Smac mimetic treatment in the presence or absence of BHA or amytal for different periods of time as indicated. For each of these different cells/cell lines, a time course that can reach 60-80% cell death of the given cell line was selected and used in the experiment. The concentrations of hTNF and Smac mimetic were 30 ng/ml and 100 nM, respectively. For macrophage treatment, 100 ng/ml mTNF was used. **e**. ROS in the cells described in (d) were measured by flow cytometry with MitoSOX. The measurements were performed at time 0 (no cell death) and the time point when cell death reached 50%. **f**. *TNFR1* KO L929 cells expressing Flag-HBD*-tTNFR1 were treated with 4-OHT+zVAD for time periods as indicated, and subjected to anti-Flag Western blotting under non-reducing condition. **g**. *RIP1* KO L929 cells expressing Flag-RIP1ΔDD-HBD* were treated with 4-OHT+zVAD for time periods as indicated, and subjected to anti-RIP1 Western blotting under non-reducing condition. **h**. *RIP3* KO L929 cells expressing Flag-RIP3-RHIM^{mut}-HBD* were treated with 4-OHT+zVAD for time periods as indicated, and subjected to anti-RIP3 Western blotting under non-reducing condition. Data shown in (d) and (e) were mean ± s.e.m of three independent experiments. *: $p<0.05$; **: $p<0.01$; ns: no significant difference. Scale bar: 15 μm. The images are representatives of pictures taken from at least 10 fields. Data shown in (f-h) are representatives of two to three independent experiments.

a

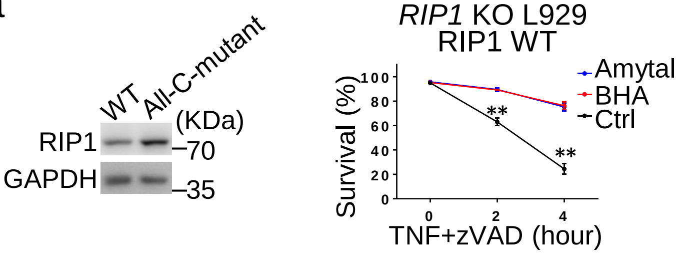

b

```
                                 34                         257        268         586
D3ZYL0|D3ZYL0_RAT     DSGGFGKVSLCFHRTHGFVILKKVY  VEDILEFCPREIISLMERCWQ  KQWKNCARKLGF
Q60855|RIPK1_MOUSE    DSGGFGKVSLCYHRSHGFVILKKVY  VEEILEYCPREIISLMERCWQ  RQWKNCARKLGF
Q13546|RIPK1_HUMAN    DSGGFGKVSLCFHRTQGLMIMKTVY  VDDITEYCPREIISLMKLCWE  KHWKNCARKLGF
H2QS79|H2QS79_PANTR   DSGGFGKVSLCFHRTQGLMIMKTVY  VDDITEYCPREIISLMKLCWE  KHWKNCARKLGF
Q3SZK6|Q3SZK6_BOVIN   DSGGFGKVCLCLHRSHGLVILKKVY  VEDIIEFCPREVIDIMRQCWE  RHWKNCARKLGF
M3WL13|M3WL13_FELCA   DSGGFGKVSLCLHRSHGLVILKKVY  VEDIIEYCPEEIISIMKQCWE  RQWKNCARKLGF
F6RUM0|F6RUM0_HORSE   DSGGFGKVSLCLNRSHELVILKKVY  VEDIIEYCPKEIISLMKHCWE  KHWKNCARKLSF
G1M741|G1M741_AILME   DSGGFGKVSLCLHRSHGLVILKKVY  VEDILEYCPEEIISIMKQCWE  KQWKNCARKLGF
G1PTZ3|G1PTZ3_MYOLU   DSGGFGKVSLCFHRSHGLVILKKVY  VNDIIEHCPKEIISLMKQCWE  RTWKNCARKLGF
G1T201|G1T201_RABIT   DSGGFGEVSLCFHRLHGLVILKKVY  VDDVAEDCPGELIRLMELCWE  KHWKNCARKLGL
H0V4W4|H0V4W4_CAVPO   DSGGFGAVSLCFHRMHGLVILKKVY  VDKIIEHCPTEIISLMTCCWQ  KQWKTCARKLGI
H0YVC2|H0YVC2_TAEGU   DAGGFGTISLCVHKKHGYVVLKKVY  IKEITDKCPVEVIDLMKQCWD  KQWKHCARKLGF
F1P4C2|F1P4C2_CHICK   DAGGYGKIYLCFHRTYGYVVLKTVY  IEEIIEKCPKEIIDLMKQCWE  KQWKHCARELGF
G3US08|G3US08_MELGA   DAGGYGKISLCLHKTYGHVVLKTVY  IEEIIEKCPQEIIDLMKQCWE  KQWKHCARKLGF
F6THC2|F6THC2_MACMU   DSGGFGKVSLCFHRTQGFVIMKTVY  VDDIIEYCPREIISLMKLCWE  KHWKNCARKLGF
                      *:**:* : ** .:      :::*.**  :.. : : ** *:* :*  **:  : ** ***:*.:
```

c

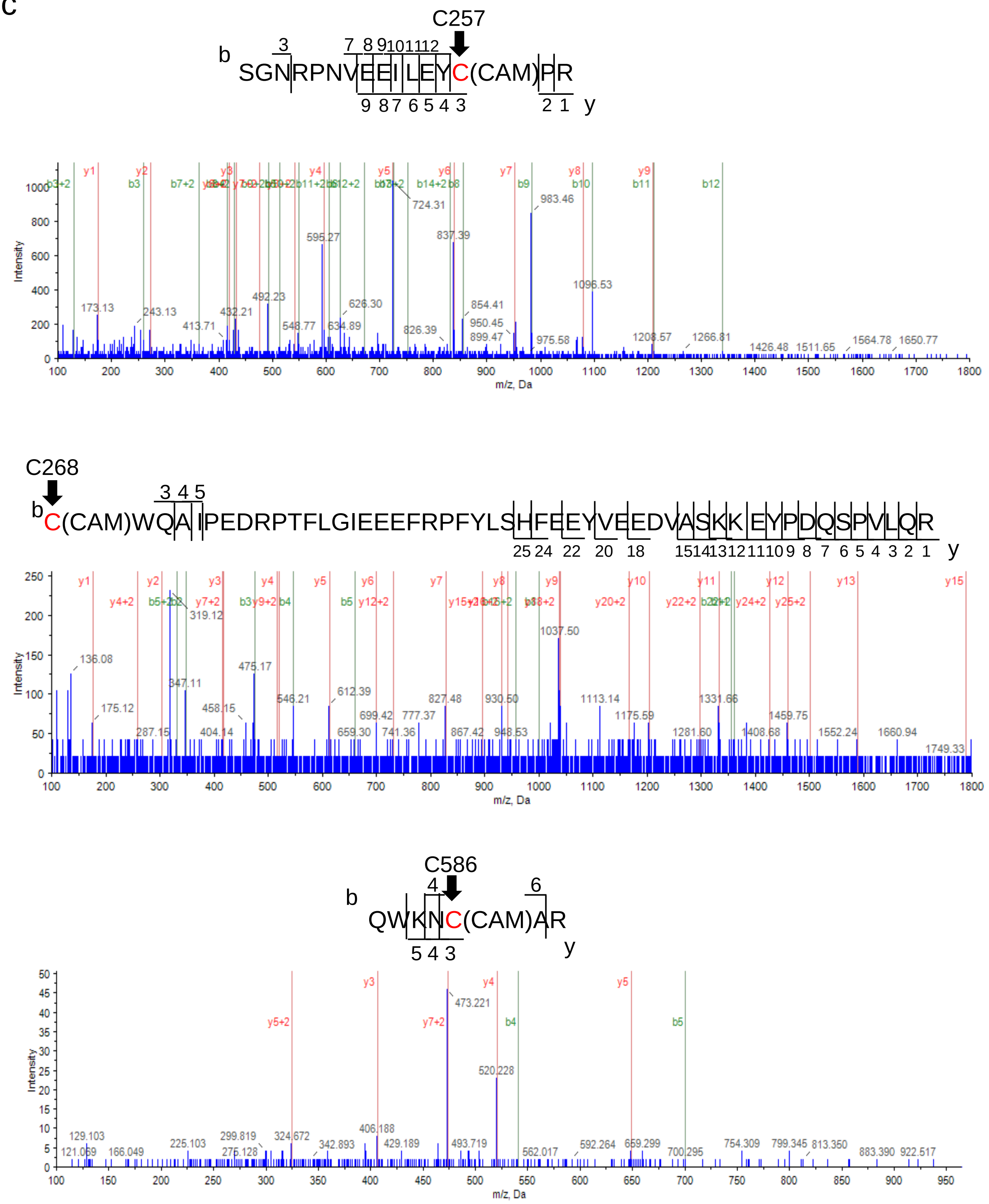

Supplementary Figure 2. Cysteine 257, 268, and 586 are targeted by ROS in necroptosis, related to Figure 2. **a**. All cysteines on RIP1 were mutated to serines. Then the mutant (All-C-mutant) was expressed in *RIP1* KO L929 cells by lentivirus. Viabilities were measured by PI exclusion after treatment of mTNF+zVAD with or without BHA/amytal at different time points as indicated. RIP1 expression levels were determined by Western blotting with anti-RIP1 antibody. **b**. Alignment of RIP1 among 15 different species using an online tool at http://www.uniprot.org/. The conserved cysteines C34, C257, C268, and C586 were highlighted in yellow. **c**. MS2 spectra of peptides containing C257, C268, and C586, respectively. The b and y product ions were labeled. The sequences of tryptic peptides containing the oxidized cysteine (in red) are shown on the top of the spectra. Data shown in (a) were mean ± s.e.m of three independent experiments. **: $p<0.01$; ns: no significant difference.

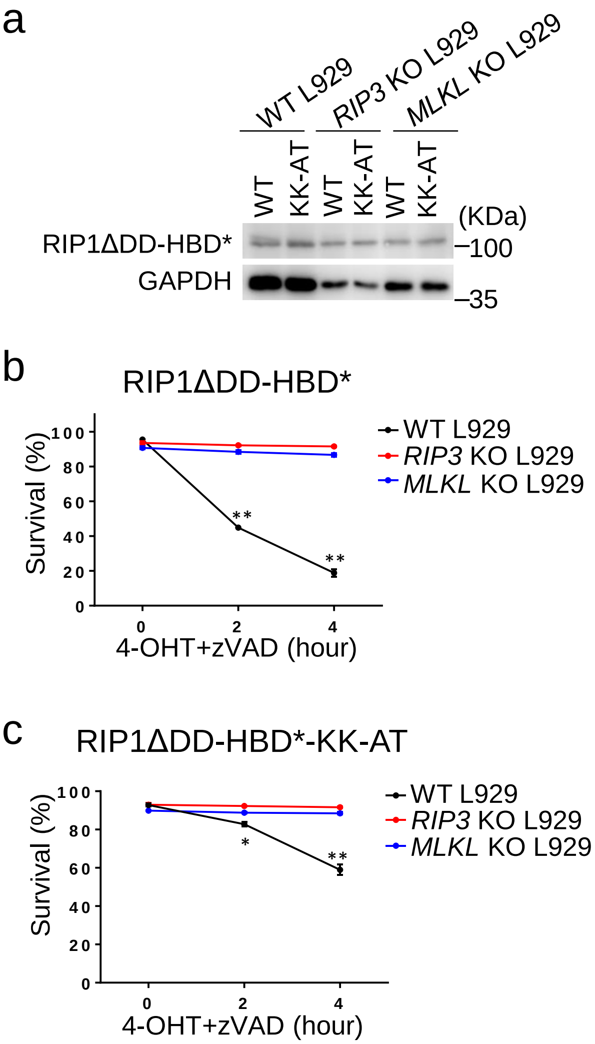
a
WT L929
RIP3 KO L929
MLKL KO L929
WT
KK-AT
WT
KK-AT
WT
KK-AT
(KDa)
RIP1ΔDD-HBD*
–100
GAPDH
–35
b
RIP1ΔDD-HBD*
Survival (%)
100
80
60
40
20
0
**
**
0
2
4
4-OHT+zVAD (hour)
WT L929
RIP3 KO L929
MLKL KO L929
c
RIP1ΔDD-HBD*-KK-AT
Survival (%)
100
80
60
40
20
0
*
**
0
2
4
4-OHT+zVAD (hour)
WT L929
RIP3 KO L929
MLKL KO L929

d

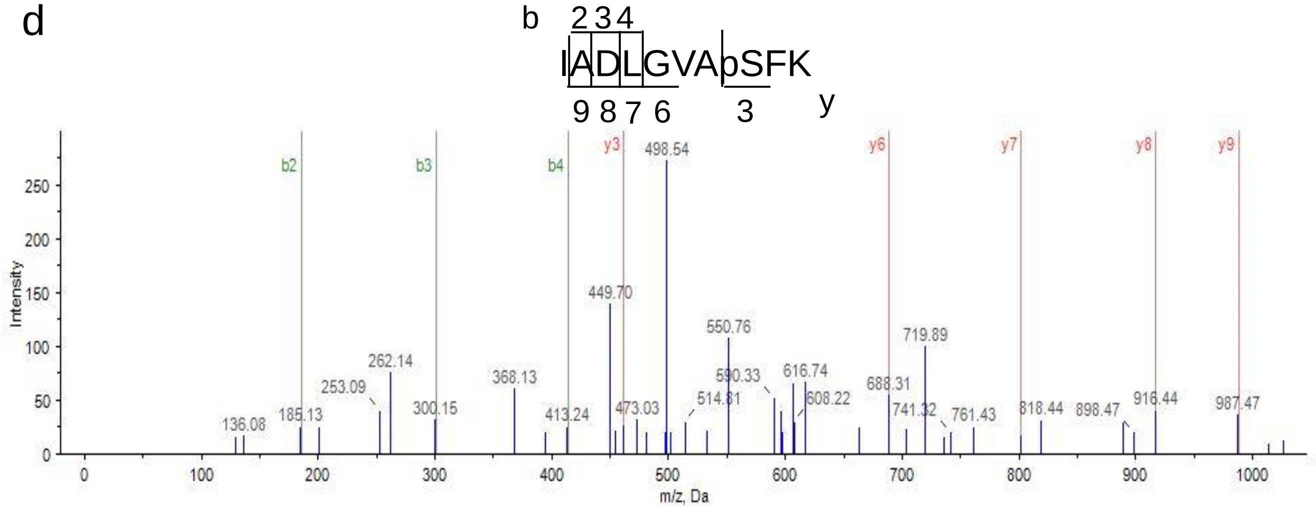

MS/MS spectrum of phosphopeptide IADLGVApSFK containing S161

e

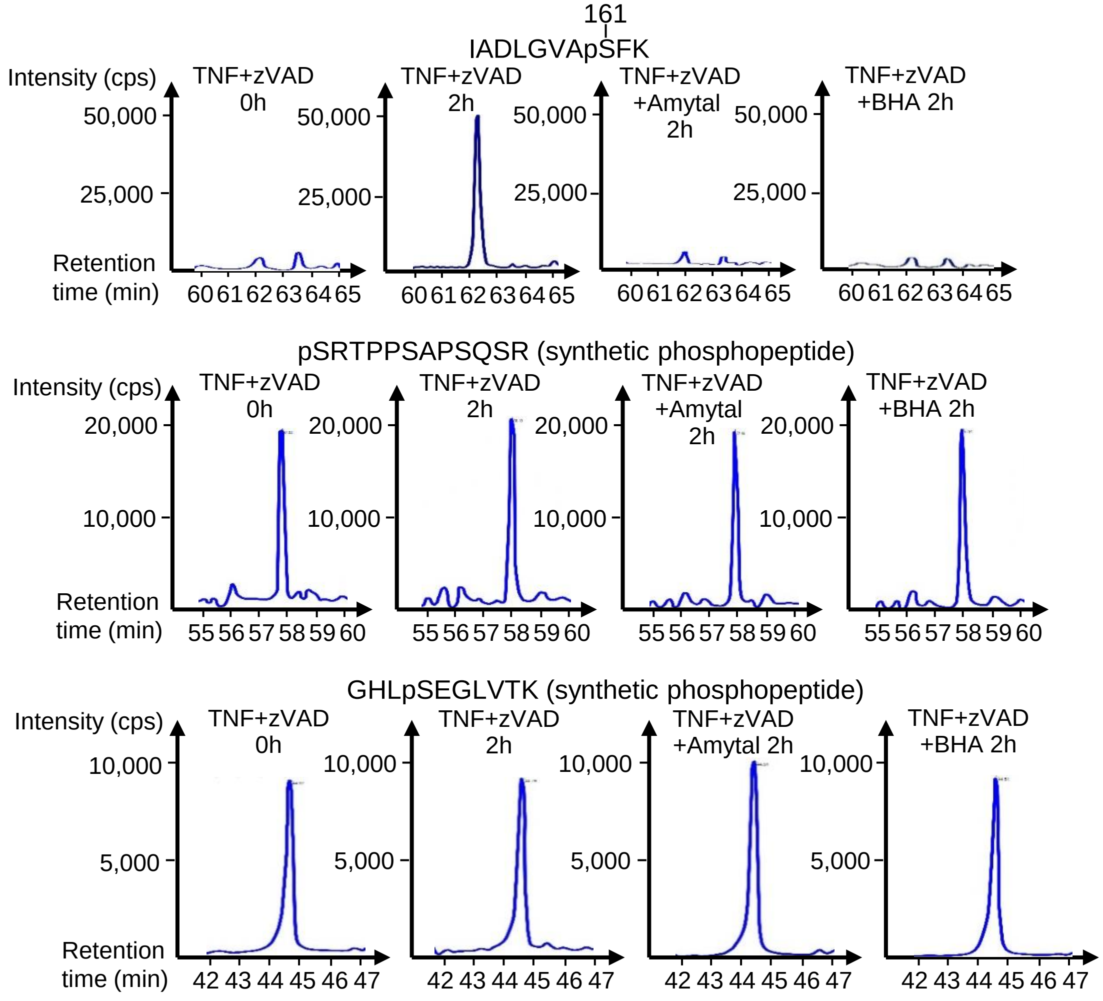

Supplementary Figure 3. RIP1 autophosphorylates S161, related to Figure 3.
a. Flag-tagged RIP1ΔDD-HBD* (WT) or RIP1ΔDD-HBD*-KK-AT (KK-AT) was expressed in WT, *RIP3* KO, and *MLKL* KO L929 cells by lentiviral vector. Their expression levels were measured by Western blotting with anti-RIP1 antibody. **b**. The cells expressing RIP1ΔDD-HBD* were treated with 4-OHT+zVAD for time periods as indicated. Viabilities of the cells were measured by PI exclusion. **c**. The same as in (b) except that the cells expressing RIP1ΔDD-HBD*-KK-AT were used. **d**. MS2 spectrum of the phosphopeptide containing S161. The b and y product ions were labeled. **e**. Falg-RIP1 reconstituted *RIP1* KO L929 cells were treated with mTNF+zVAD for 0 or 2 hours in the presence or absence of BHA or amytal. RIP1 was immunoprecipitated by anti-Flag M2 beads. The IMAC-enriched phosphopeptides were subjected to MS analyses on RIP1 S161 and two synthetic phosphopeptides were added into the samples as internal controls for IMAC enrichment. Ions with an m/z value representing RIP1 S161 or two synthetic phosphopeptides were monitored by MS, and MS2 XIC (extracted-ion chromatogram) peaks in each sample were shown. Data shown in (b-c) were mean ± s.e.m of two independent experiments. *: p<0.05; **: p<0.01.

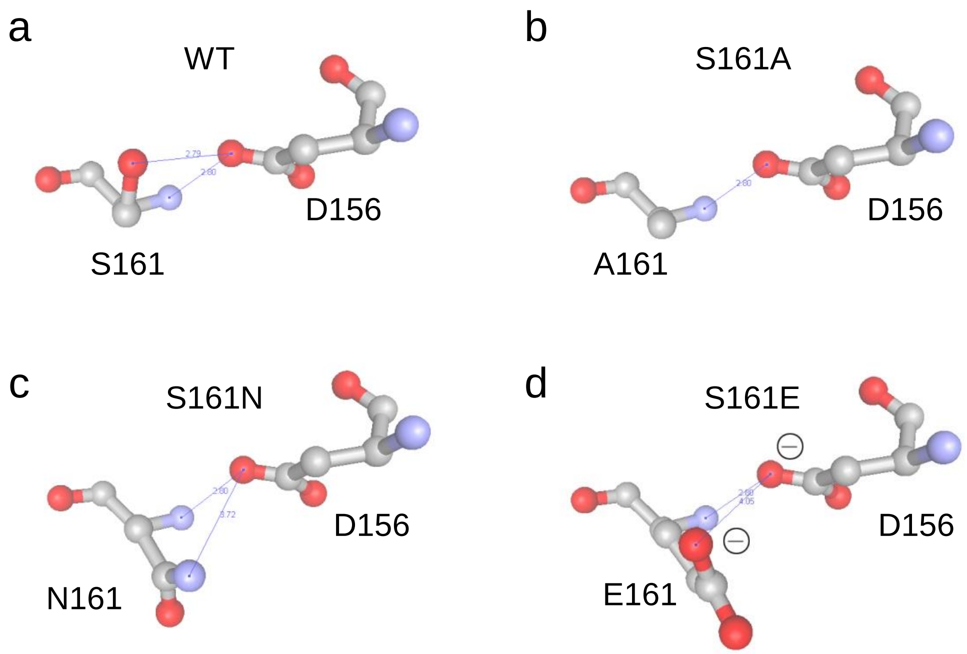

Supplementary Figure 4. 3D modeling of interaction between S161 and D156 of RIP1, related to Figure 4.
a. 3D configuration modeling of S161 and D156 was achieved by the software Coot9 and VectorNTI based on the crystal structure of RIP1 (PDB No. 4ITH). Red balls, blue balls and gray parts stand for oxygen atoms, nitrogen atoms and carbon skeleton respectively. Lines and numbers colored in blue represent the distance between two atoms to form hydrogen bonds, and the unit is Å. **b-d**. S161 was replaced by alanine, asparagine and glutamic acid respectively, and interaction models were predicted and rebuilt as described above.

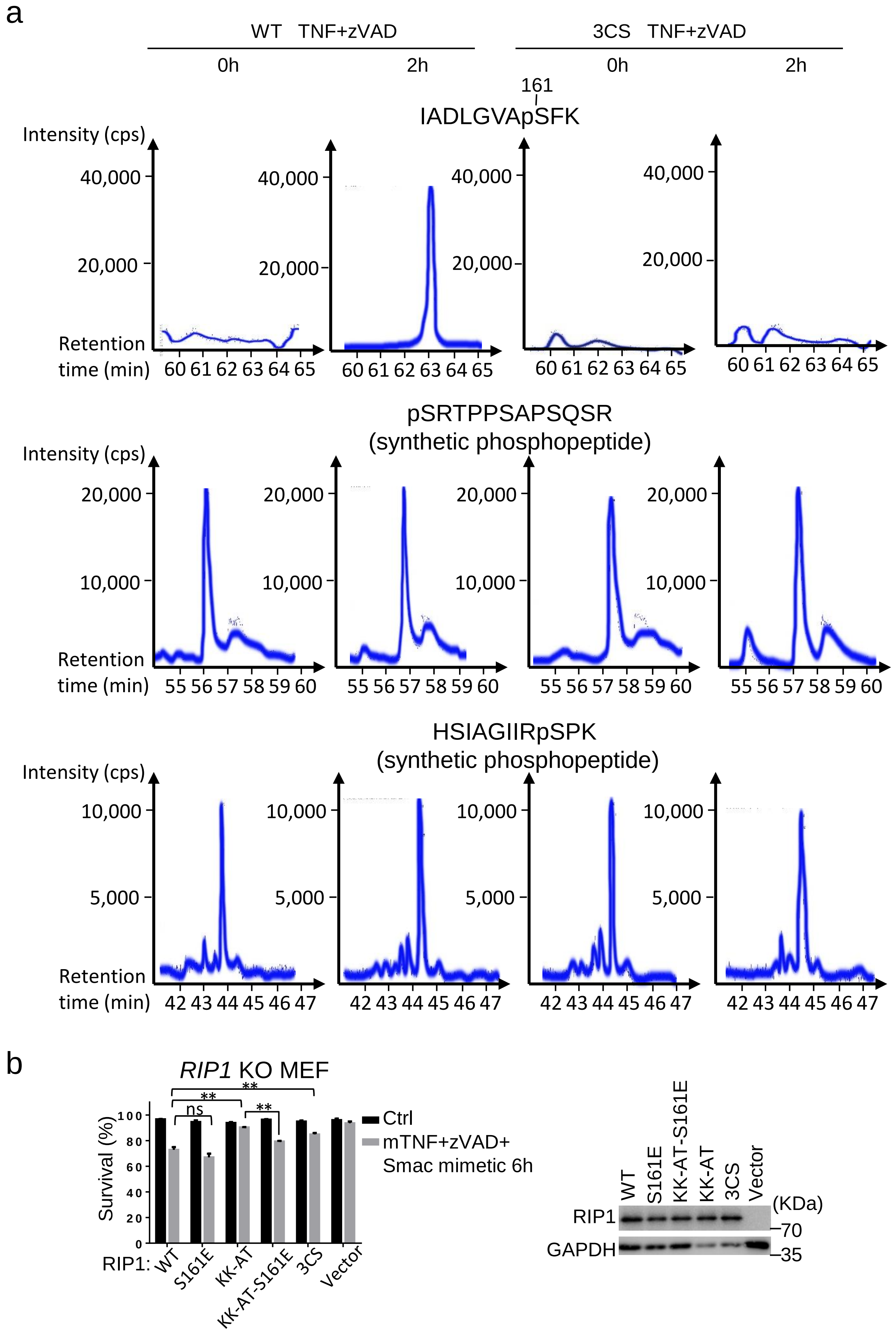

a
WT TNF+zVAD
3CS TNF+zVAD
0h
2h
0h
2h
161
IADLGVApSFK
Intensity (cps)
40,000
20,000
Retention time (min)
60 61 62 63 64 65
pSRTPPSAPSQSR
(synthetic phosphopeptide)
20,000
10,000
55 56 57 58 59 60
HSIAGIIRpSPK
(synthetic phosphopeptide)
10,000
5,000
42 43 44 45 46 47
b
RIP1 KO MEF
Survival (%)
ns
**
Ctrl
mTNF+zVAD+
Smac mimetic 6h
RIP1:
WT
S161E
KK-AT
KK-AT-S161E
3CS
Vector
(KDa)
RIP1
GAPDH
70
35

c

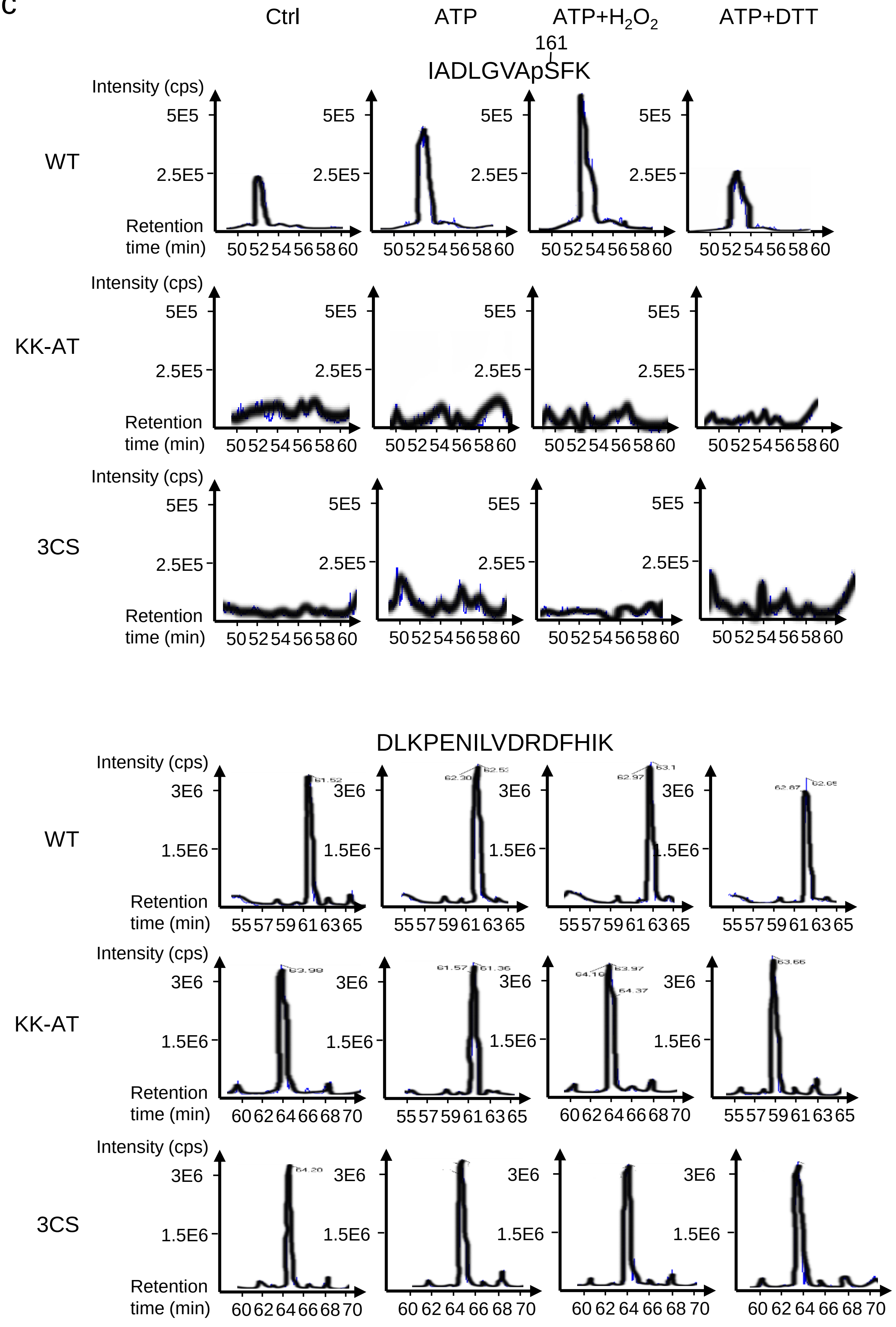

Ctrl
ATP
ATP+H2O2
ATP+DTT
161
IADLGVApSFK
Intensity (cps)
5E5
2.5E5
Retention time (min)
50 52 54 56 58 60
WT
KK-AT
3CS
DLKPENILVDRDFHIK
3E6
1.5E6
55 57 59 61 63 65
60 62 64 66 68 70

Supplementary Figure 5. Cysteine oxidation in RIP1 affects its S161 phosphorylation, related to Figure 5.
a. Flag-RIP1 (WT) or Flag-RIP1-3CS (3CS) reconstituted *RIP1* KO L929 cells were treated with mTNF+zVAD for 0 or 2 hours. RIP1 was immunoprecipitated by anti-Flag M2 beads. The IMAC-enriched phosphopeptides were subjected to MS analyses on RIP1 S161 and two synthetic phosphopeptides were added into the samples as internal controls for IMAC enrichment. Ions with an m/z value representing RIP1 S161 or two synthetic phosphopeptides were monitored by MS, and MS2 XIC (extracted-ion chromatogram) peaks in each sample were shown. **b**. *RIP1* KO MEF cells were reconstituted with WT, S161E, KK-AT, KK-AT-S161E, 3CS RIP1 or control vector. The protein expression levels were determined by Western blotting with anti-RIP1 antibody. Viabilities of the cells treated with mTNF+zVAD+Smac mimetic for 6 hours were measured by PI exclusion. **: $p<0.01$; ns: no significant difference. Data shown were mean $\pm$ s.e.m of three independent experiments. **c**. *In vitro* autophosphorylation of purified Flag-tagged WT, KK-AT, or 3CS RIP1 was measured by MS. Products after kinase reactions were TCA precipitated and subjected to MS analyses on RIP1 S161-containing tryptic peptide. RIP1 tryptic peptide DLKPENILVDRDFHIK was selected as internal control for quantitation. Ions with an m/z value representing phospho-S161-containing peptide or peptide DLKPENILVDRDFHIK were monitored by MS, and MS2 XIC (extracted-ion chromatogram) peaks in each sample were shown. Ctrl means no kinase reaction was conducted because of no ATP. ATP, ATP+H_2O_2, and ATP+DTT mean that kinase reaction was performed using purified proteins pretreated with nothing, H_2O_2, and DTT, respectively.

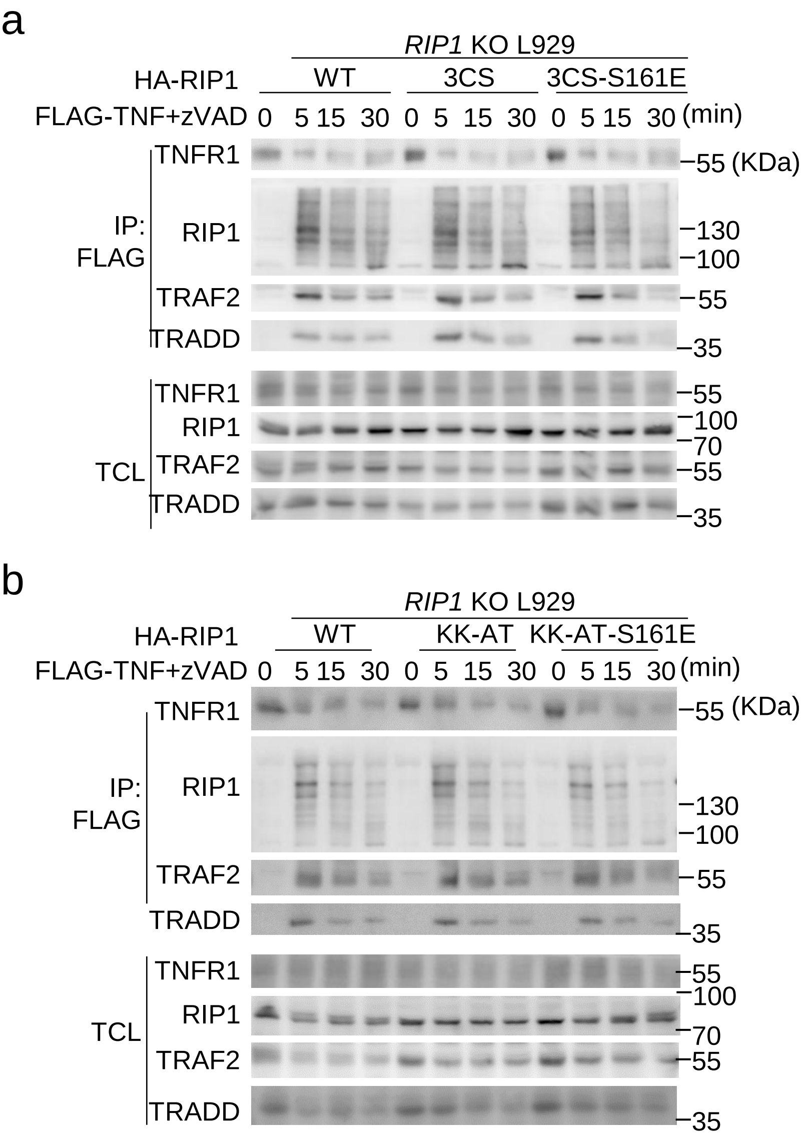
a
RIP1 KO L929
HA-RIP1
WT
3CS
3CS-S161E
FLAG-TNF+zVAD 0 5 15 30 0 5 15 30 0 5 15 30 (min)
IP: FLAG
TNFR1
RIP1
TRAF2
TRADD
TCL
TNFR1
RIP1
TRAF2
TRADD
55 (KDa)
130
100
55
35
55
100
70
55
35
b
RIP1 KO L929
HA-RIP1
WT
KK-AT
KK-AT-S161E
FLAG-TNF+zVAD 0 5 15 30 0 5 15 30 0 5 15 30 (min)
IP: FLAG
TNFR1
RIP1
TRAF2
TRADD
TCL
TNFR1
RIP1
TRAF2
TRADD
55 (KDa)
130
100
55
35
55
100
70
55
35

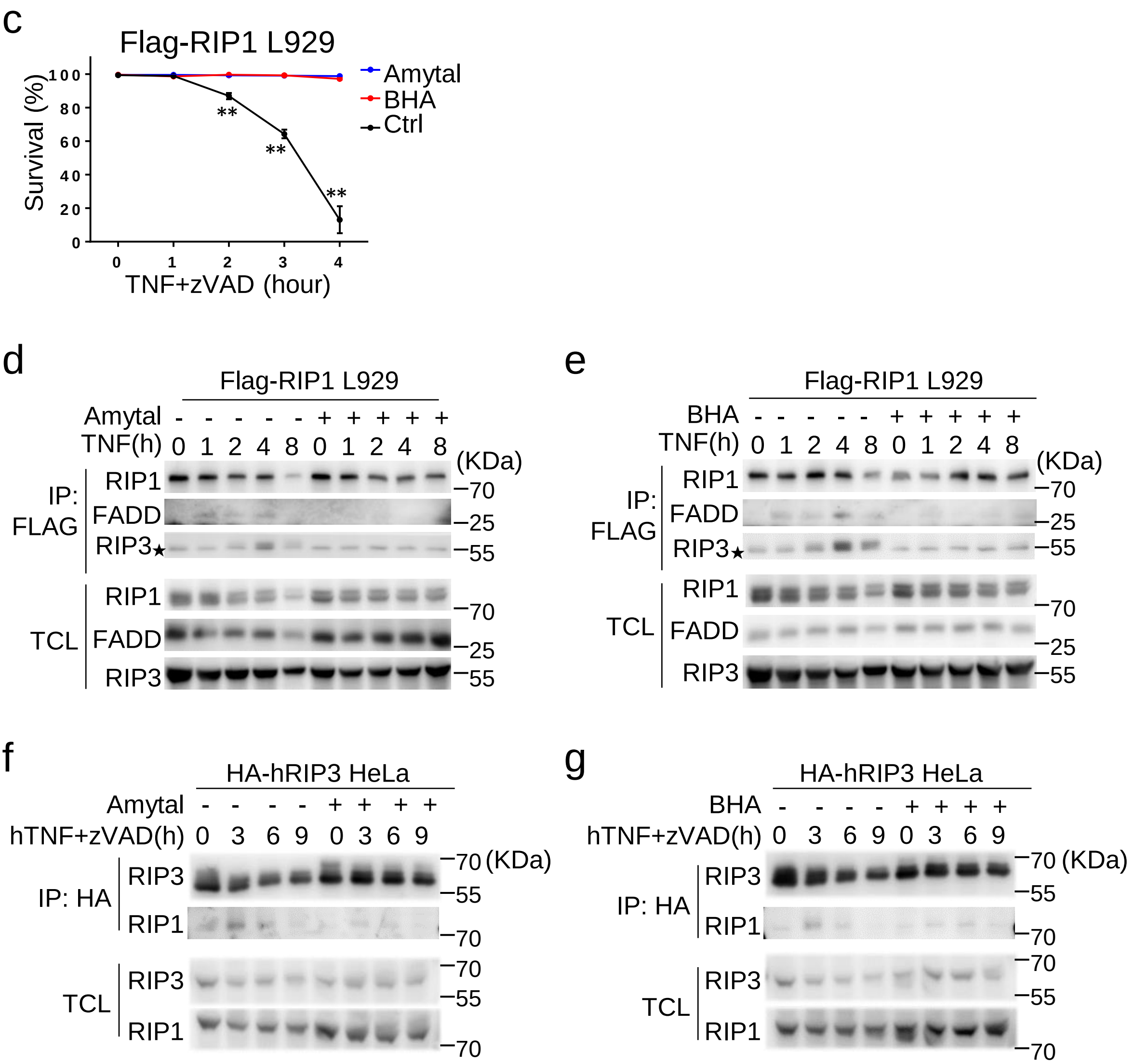

Supplementary Figure 6. S161 phosphorylation has no effect on complex I formation, related to Figure 6 and 7. **a**. *RIP1* KO L929 cells reconstituted with WT, 3CS, or 3CS-S161E RIP1 were treated with Flag-TNF (10 ng/ml) +zVAD for time periods as indicated. Cells were then lysed and Flag-TNF was immunoprecipitated with anti-FLAG M2 beads. Samples were applied to Western blotting with antibodies as indicated to detect complex I components. **b**. The same as in (a) except that *RIP1* KO L929 cells reconstituted with WT, KK-AT, or KK-AT-S161E RIP1 were used. **c**. Viabilities of Flag knock in L929 (Flag-RIP1 L929) were determined at different time points as indicated after mTNF+zVAD treatment with or without BHA or amytal. Data shown were mean ± s.e.m of three independent experiments. **: *p*<0.01. **d-e**. Flag-RIP1 L929 cells were treated with mTNF alone for the indicated time periods with or without BHA or amytal. Cell lysates were subjected to immunoprecipitation with anti-FLAG M2 beads and then Western blotting with antibodies as indicated. ★: nonspecific band. **f-g**. HeLa cells were infected with HA-hRIP3 lentivirus for 24 hours, and stimulated with hTNF (30 ng/ml) +zVAD for the indicated time periods in the presence or absence of BHA or amytal, and then lysed for immunoprecipitation with anti-HA beads and Western blotting with anti-RIP1 and anti-human RIP3 antibodies. Data shown in (a-b, d-g) are representatives of two to three independent experiments.

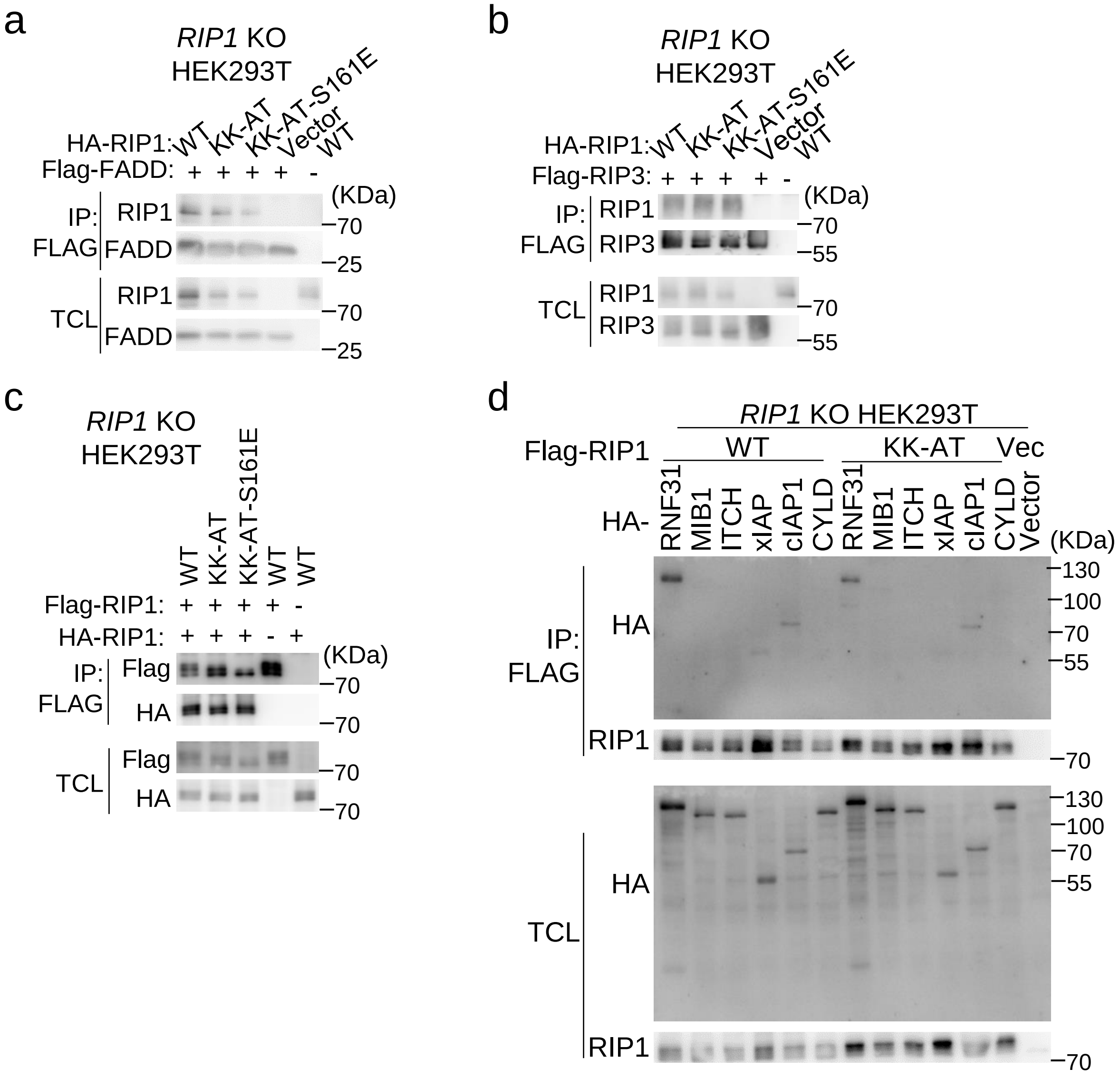

Supplementary Figure 7. Analyzing the interaction of RIP1 and its autophosphorylation-related mutants with the components of necrosome by co-expression assay, related to Figure 6 and 7.

a-c. HA-RIP1 (WT) and its mutants were overexpressed in *RIP1* KO HEK293T cells, respectively, together with Flag-FADD, Flag-RIP3 or Flag-RIP1. 24 hours after transfection, cells were lysed and subjected to co-immunoprecipitation with anti-FLAG M2 beads and then Western blotting analyses with anti-FADD, anti-RIP1, anti-RIP3, anti-Flag, anti-HA antibodies as indicated. **d**. *RIP1* KO HEK293T cells were co-expressed with HA-tagged ubiquitination-related proteins and Flag-RIP1-WT or Flag-RIP1-KK-AT. Co-immunoprecipitation with M2 beads and Western blotting for HA and RIP1 were performed. These UB-related proteins include RNF31, MIB1, ITCH, xIAP, cIAP1 and CYLD. Data shown in (a-d) are representatives of two to three independent experiments.

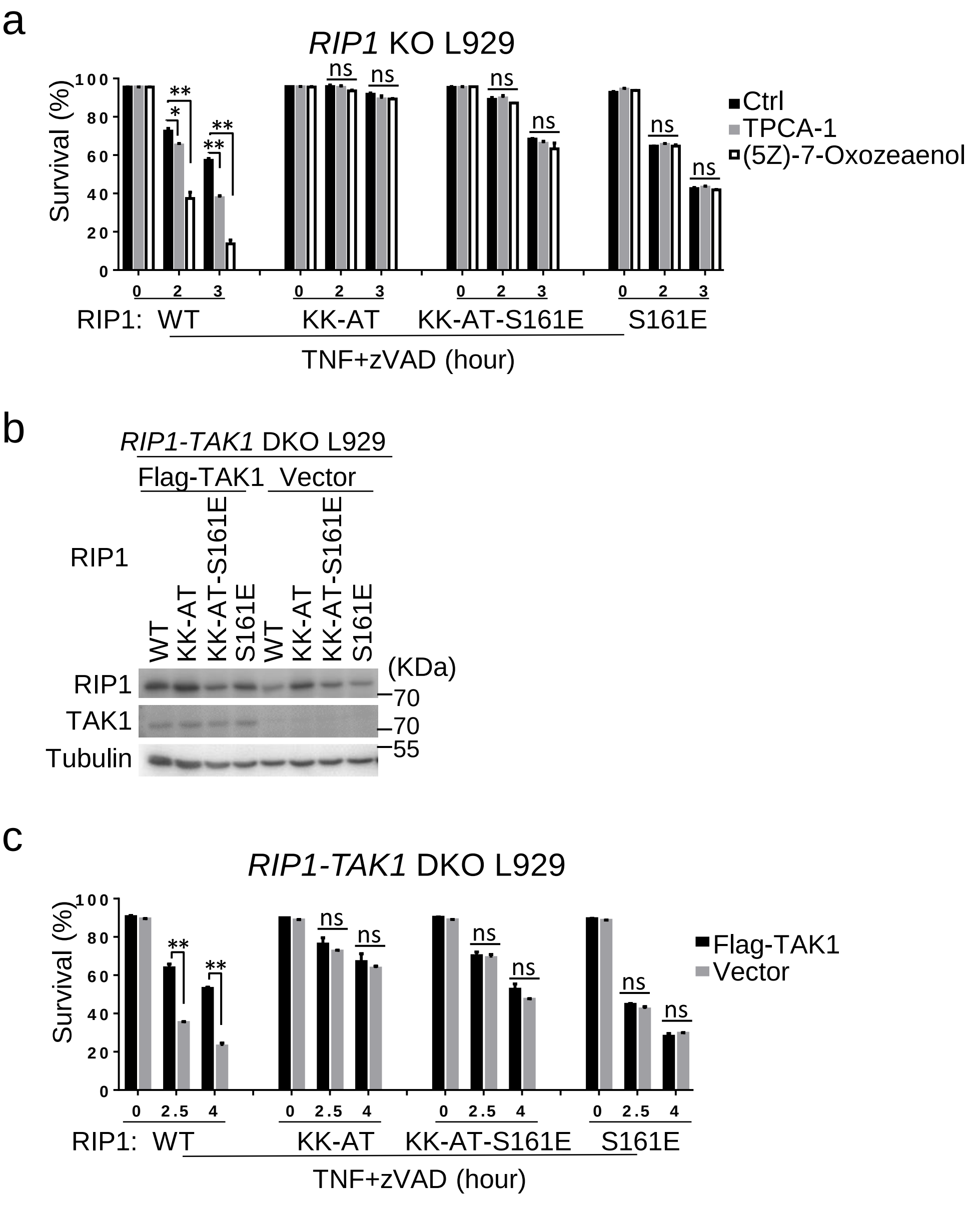

Supplementary Figure 8. S161 phosphomimetic RIP1 mutant is insensitive to IKKα/IKKβ inhibition of necroptosis

a. *RIP1* KO L929 cells were reconstituted with WT, KK-AT, KK-AT-S161E and S161E RIP1, respectively. Viabilities were measured by PI exclusion at different time points after mTNF+zVAD treatment with or without TPCA-1 (40 μM) or (5Z)-7-Oxozeaenol (1 μM). **b**. *RIP1-TAK1* DKO L929 cells were co-expressed with TAK1 and RIP1-WT or its mutants as indicated. Western blotting was carried out to measure the expression level of RIP1 and TAK1 24 hours after lentivirus infection. Tubulin was used as a loading control. **c**. Cells obtained above were treated with mTNF+zVAD for different periods of time. Viabilities were measured by PI exclusion. Data in (a), (c) represented the mean ± s.e.m of two independent experiments.

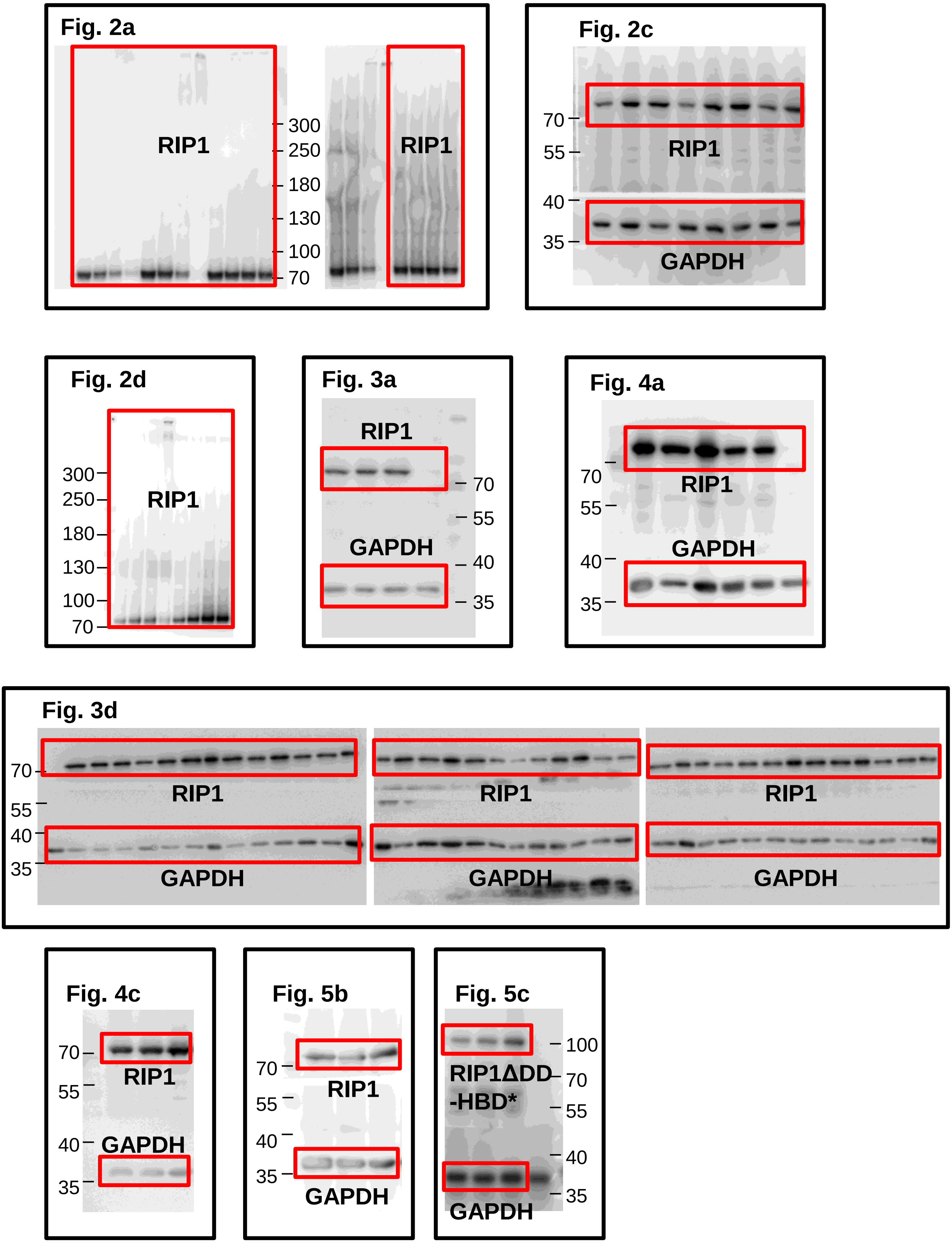

Supplementary Figure 9. Uncropped images of blots in Figure 2-6 and Supplementary Figure 1-3 and 5-8.

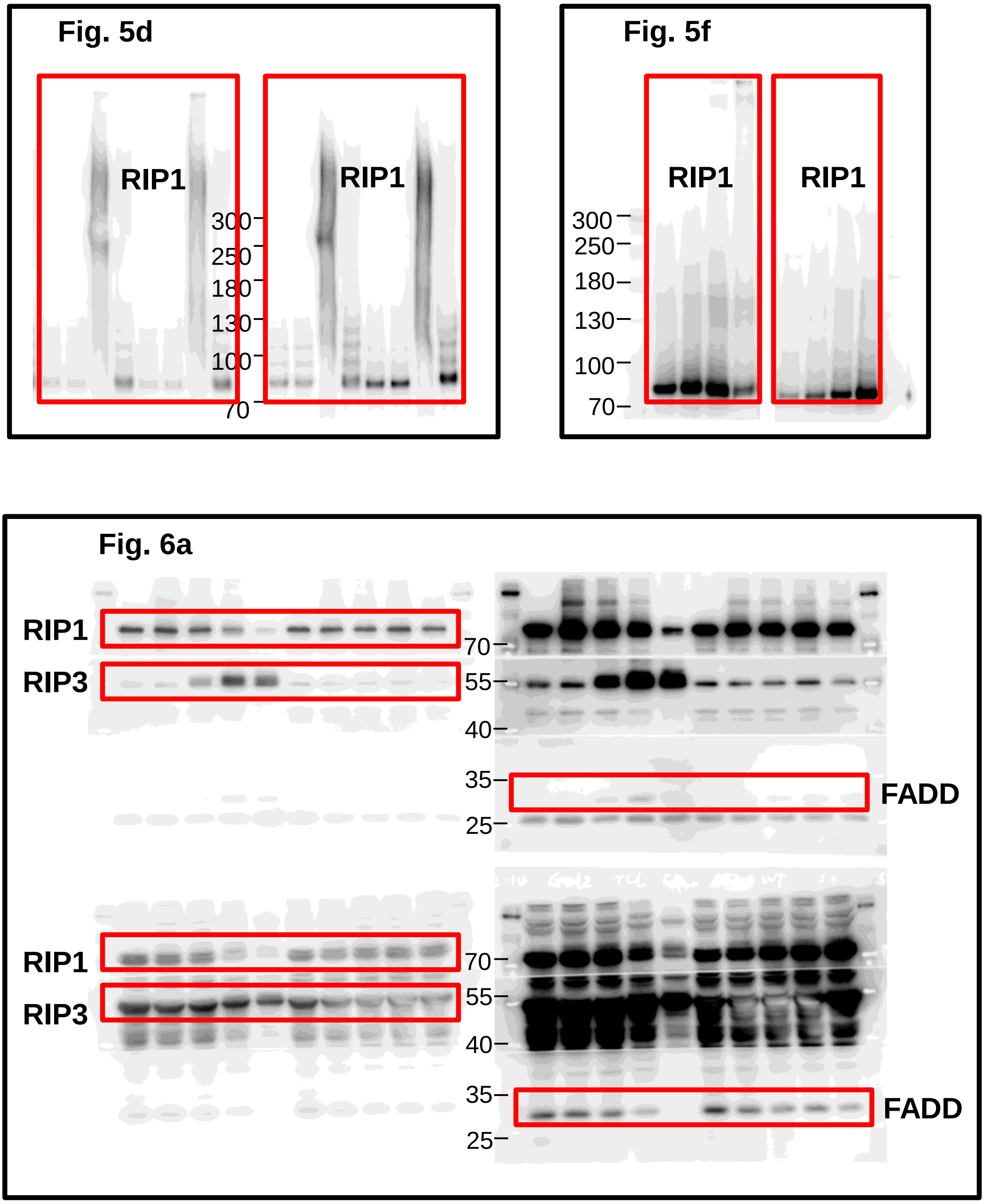

Supplementary Figure 9. Uncropped images of blots in Figure 2-6 and Supplementary Figure 1-3 and 5-8.

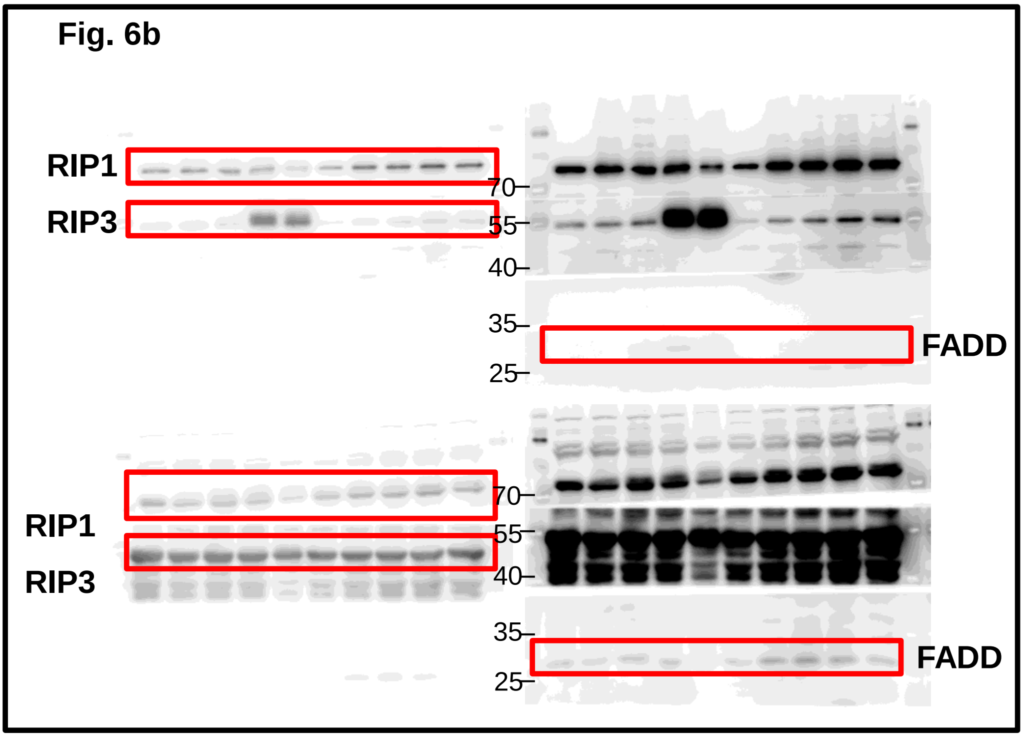

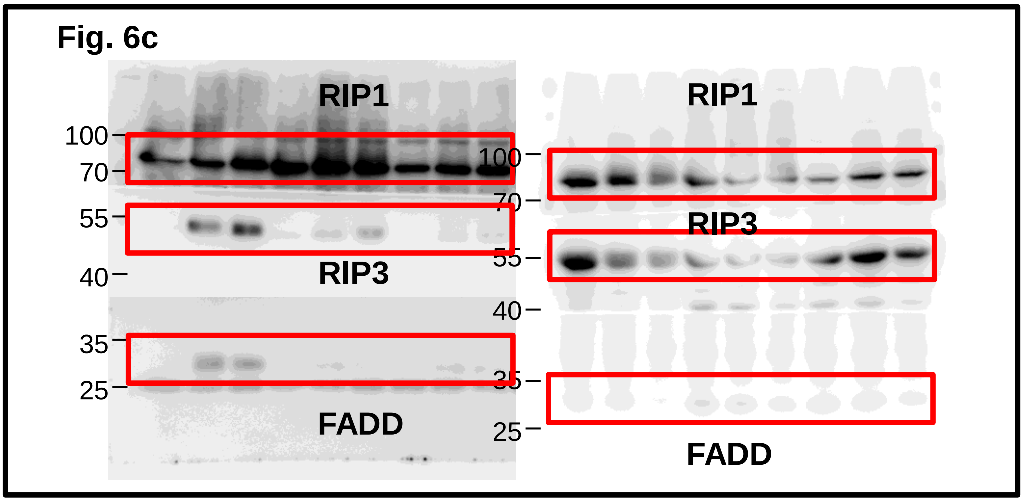

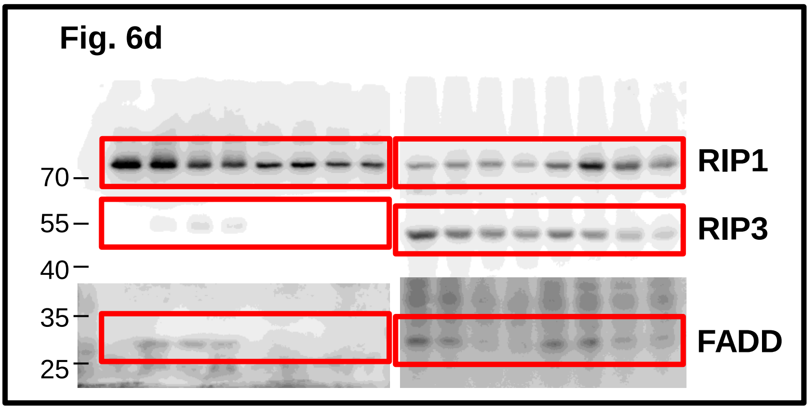

Supplementary Figure 9. Uncropped images of blots in Figure 2-6 and Supplementary Figure 1-3 and 5-8.

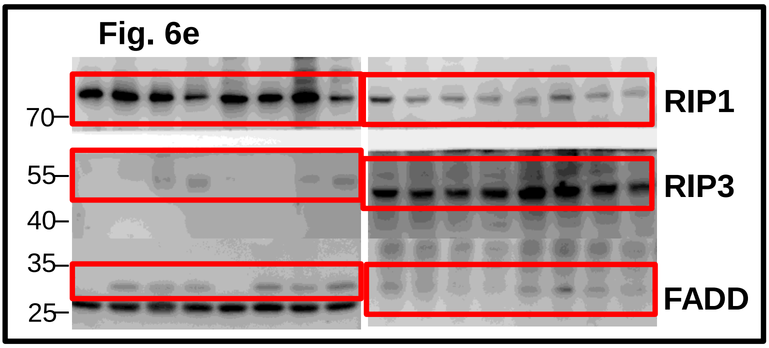

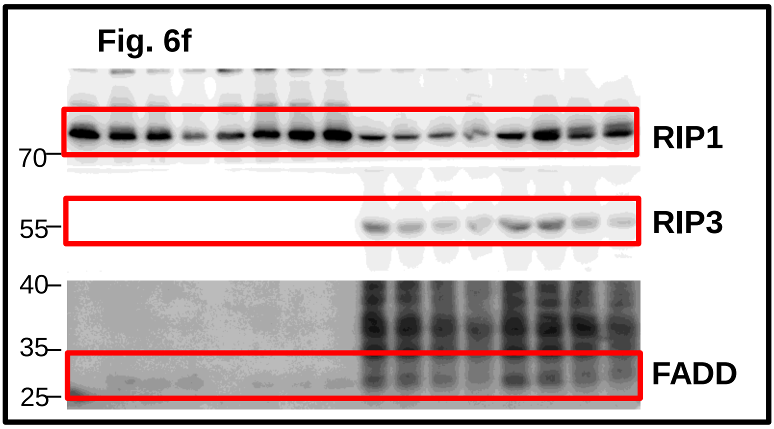

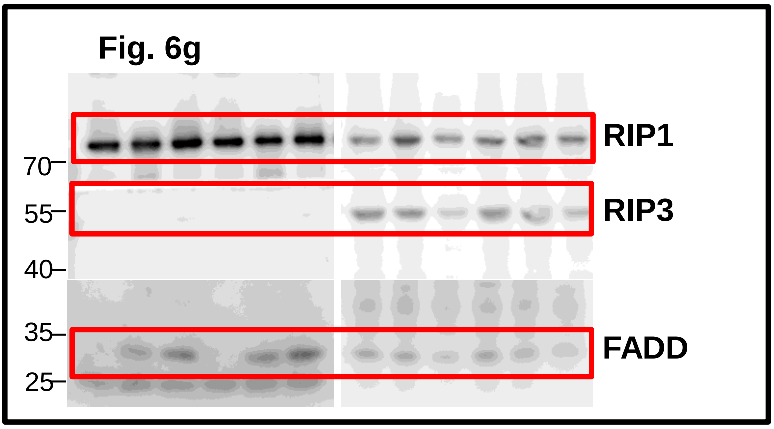

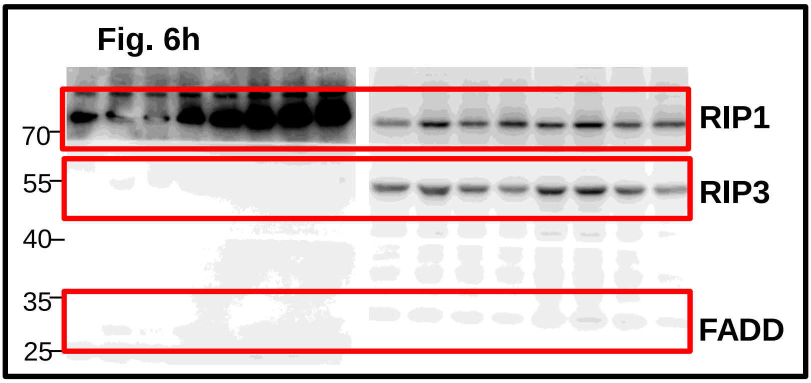

Supplementary Figure 9. Uncropped images of blots in Figure 2-6 and Supplementary Figure 1-3 and 5-8.

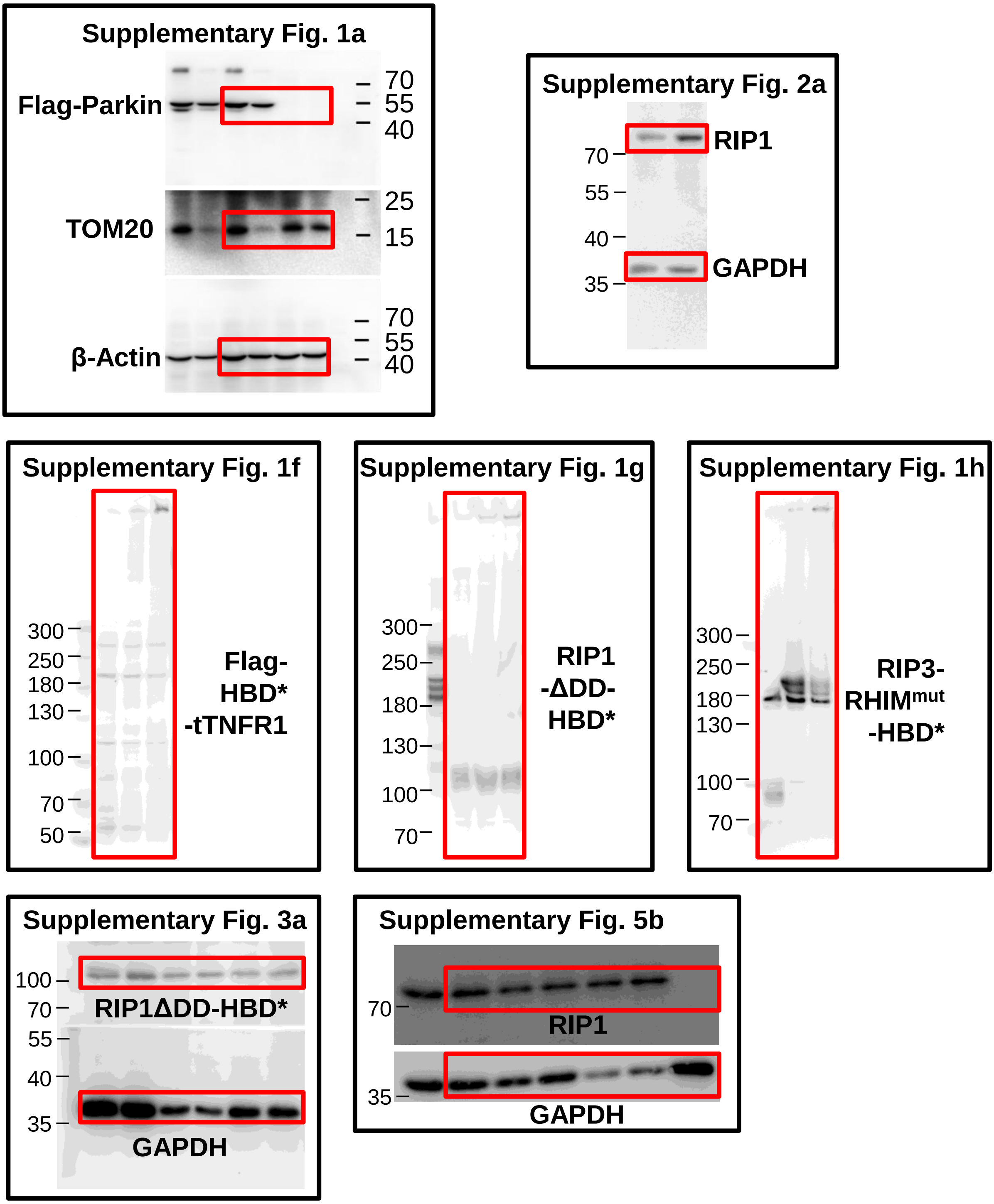

Supplementary Figure 9. Uncropped images of blots in Figure 2-6 and Supplementary Figure 1-3 and 5-8.

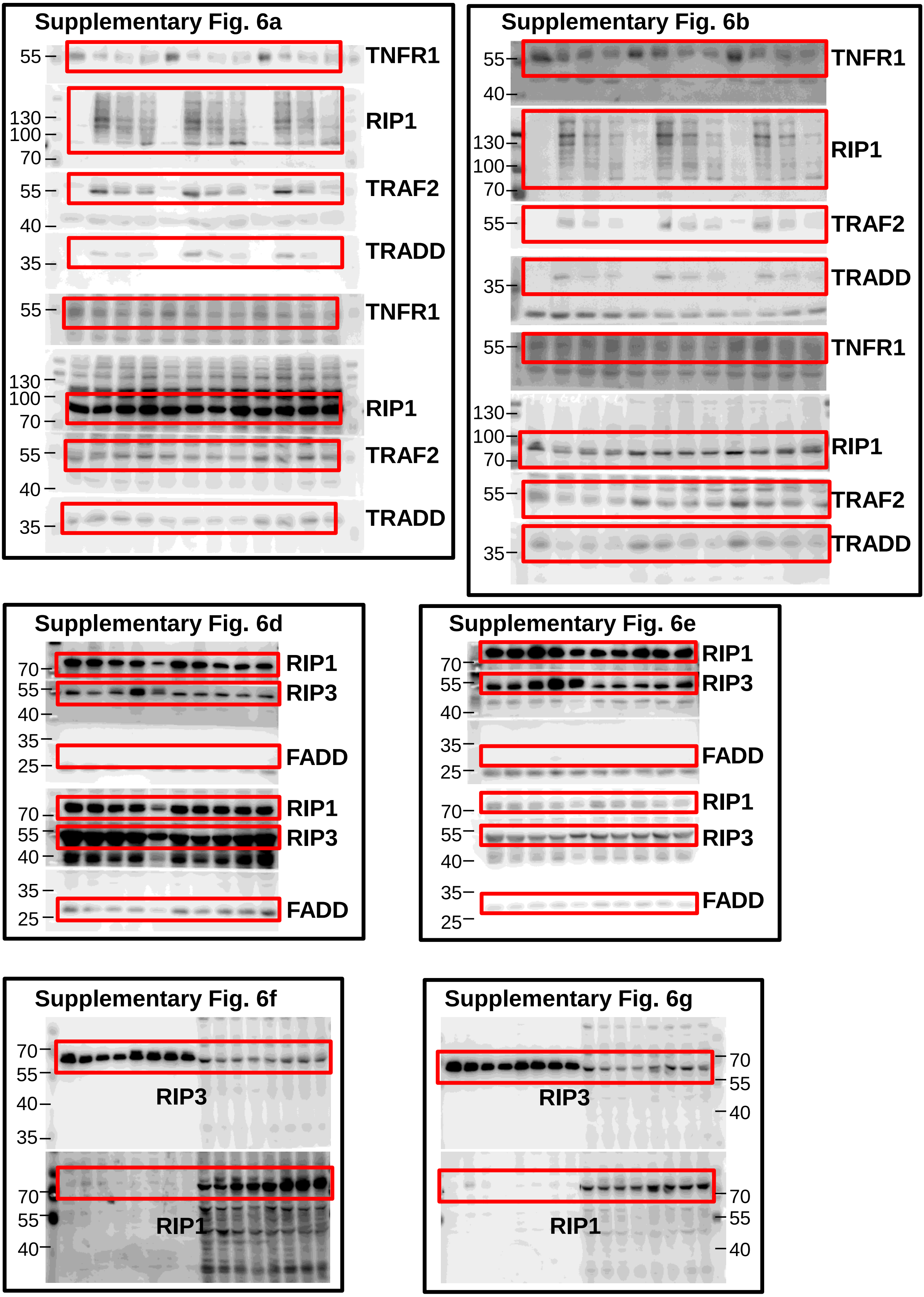

Supplementary Figure 9. Uncropped images of blots in Figure 2-6 and Supplementary Figure 1-3 and 5-8.

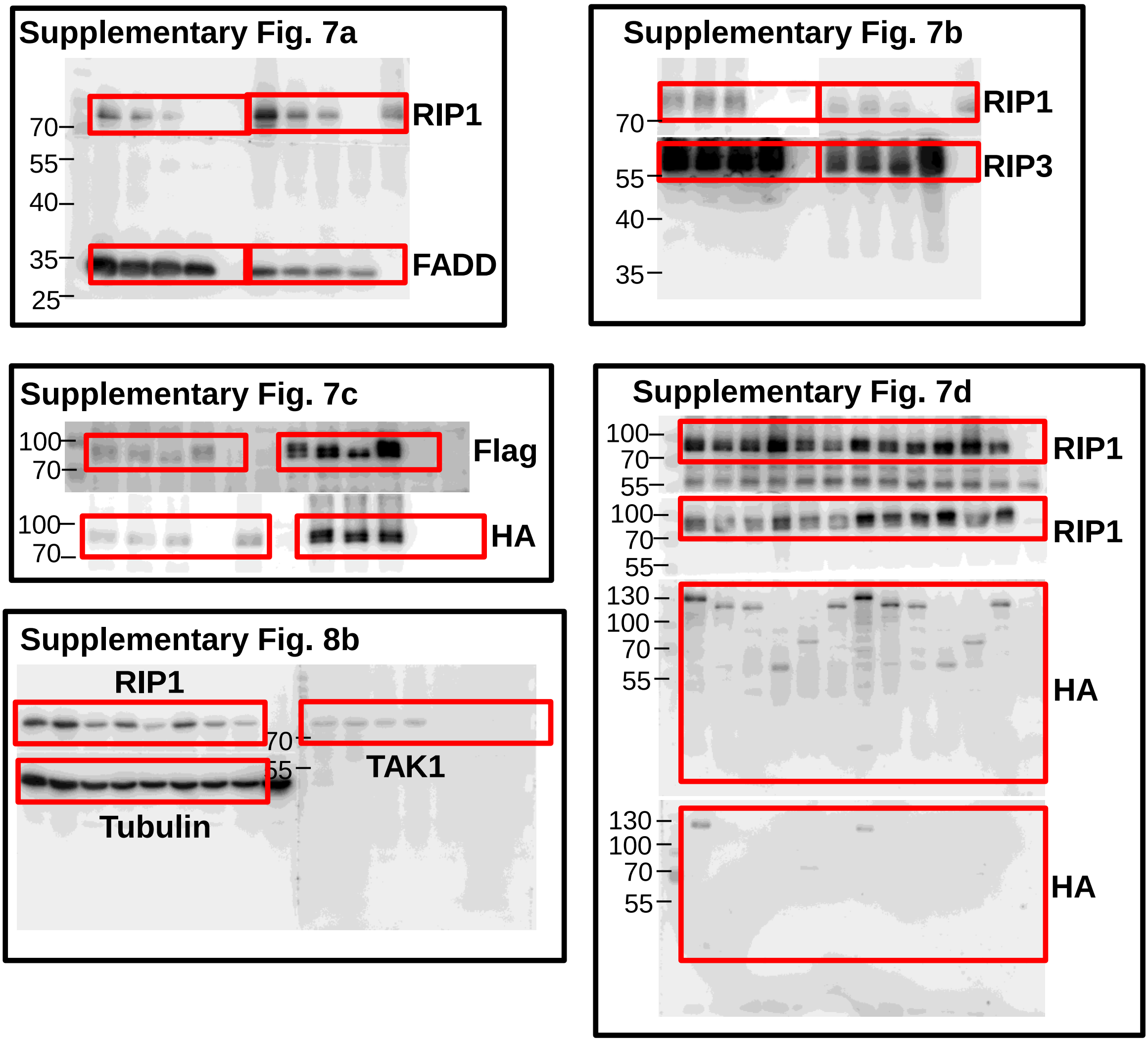

Supplementary Figure 9. Uncropped images of blots in Figure 2-6 and Supplementary Figure 1-3 and 5-8.
